# Treating human cancer by targeting EZH2

**DOI:** 10.1016/j.gendis.2024.101313

**Published:** 2024-04-25

**Authors:** Mengfei Xu, Chunyan Xu, Rui Wang, Qing Tang, Qichun Zhou, Wanyin Wu, Xinliang Wan, Handan Mo, Jun Pan, Sumei Wang

**Affiliations:** a The Second Clinical Medical College, Guangzhou University of Chinese Medicine, Guangzhou, Guangdong 510120, China; b Department of Oncology, Clinical and Basic Research Team of TCM Prevention and Treatment of NSCLC, Guangdong Provincial Hospital of Chinese Medicine, The Second Clinical College of Guangzhou University of Chinese Medicine, Guangdong Provincial Key Laboratory of Clinical Research on Traditional Chinese Medicine Syndrome, State Key Laboratory of Dampness Syndrome of Chinese Medicine, Guangzhou, Guangdong 510120, China; c Department of Urology, The Second Affiliated Hospital of Guangzhou University of Chinese Medicine, Guangzhou, Guangdong 510120, China

**Keywords:** Cancer, Epigenetic modification, EZH2, EZH2 inhibitor, H3K27me3

## Abstract

Enhancer of zeste homolog 2 (EZH2), an epigenetic regulator that primarily inhibits downstream gene expression by tri-methylating histone H3, which is usually overexpressed in tumors and participates in many processes such as tumor occurrence and development, invasion, migration, drug resistance, and anti-tumor immunity as an oncogene, making it an important biomarker in cancer therapy. Collectively, several transcription factors and RNAs cooperate to facilitate the elevated expression of EZH2 in cancer. Although the significance of blocking EZH2 in cancer for inhibiting cancer progression is widely recognized, the clinical application of EZH2 inhibitors continues to encounter numerous challenges. In this review, drawing upon our comprehensive understanding of the factual underpinnings of EZH2's role in cancer, we aim to clarify the crucial importance of targeting EZH2 in cancer treatment. Furthermore, we summarize the current research landscape surrounding targeted EZH2 inhibitors and offer insights into potential future applications of these inhibitors.

## Introduction

Cancer, a grave illness that poses a constant threat to human health, has consistently garnered significant attention from researchers. Many patients seek medical attention only when their tumors have reached the late stages, as the early stages often do not exhibit specific symptoms, thereby posing a challenge in diagnosing cancer during the early stages of tumor progression. Although surgical resection can effectively alleviate symptoms in patients, it is not suitable for all patients, and growing evidence indicates that surgical resection may facilitate the metastatic seeding of tumor cells.^1^ Owing to challenges like tumor heterogeneity and drug resistance to chemotherapy, however, the clinical outcomes of traditional cancer mono-chemotherapy frequently fall short of expectations.^2^ Enhancer of zeste homolog 2 (EZH2), an epigenetic regulator frequently overexpressed in cancer, plays a crucial role in various processes including cancer initiation and progression, invasion and migration, drug resistance, and anti-tumor immunity, thus serving as a significant biomarker in cancer therapy.^3^ Given the myriad of effects exerted by EZH2, targeting it may emerge as a powerful therapeutic agent for halting cancer progression and addressing existing challenges related to cancer treatment. On January 23, 2020, the biopharmaceutical company Epizyme announced that the Food and Drug Administration would accelerate the approval of tazveraik (tazemetostat), which was also the first EZH2 inhibitor approved for marketing. This has also further aroused people's attention to targeting EZH2 in cancer. As the diverse functions of EZH2 continue to be uncovered, it is imperative to have a precise understanding of its intricate and diverse roles to effectively target it in cancer therapy. Our work summarizes the structure of EZH2, explains the mechanisms underlying its elevated expression in cancer, and delineates its role in cancer progression.

## Structure of EZH2

EZH2, a specific histone-lysineN-methyltransferase encoded by EZH2 gene, which belongs to polycomb group proteins, is a catalytic component of polycomb repressive complex 2 (PRC2) and can specifically mediate trimethylated histone H3 at Lys 27 (H3K27me3) histone methylation modification. Its gene is located on the long arm 7q35 of the human chromosome, contains 20 exons, and encodes 746 amino acid residues. EZH2 contains several domains: SET conserved domain, cysteine-rich domain, and ncRBD domain. The C-terminal SET catalyzes H3K27me3.^4^ This domain silences target genes and participates in various biological functions, such as cell cycle, cell proliferation, and cell differentiation. Cysteine-rich domain and ncRBD domain are necessary for interaction with other PRC2 components and regulatory proteins.^5^

## Action mode of EZH2 in human cancer

### EZH2 silences gene expression through PRC2-dependent H3K27me3

Epigenetic modification, which encompasses histone modification, DNA methylation, chromatin remodeling, and the regulation of noncoding RNA, denotes alterations in gene expression and function that occur without any modifications to the DNA sequence. Furthermore, this process has been recognized and established as a significant driver of tumorigenesis.^6^

As a subunit of PRC2, EZH2 catalyzes H3K27me3 in the nucleus while polycomb repressive complex 1 (PRC1) binds to monoubiquitinated histone H2A at lysine 119 and H3K27me3. This complex mediates chromatin compaction followed by transcriptional repression of downstream genes and then involves in maintaining the characteristics of stem cells, regulating gene expression, cell cycle, cell differentiation, and the development of tissues and organs.^7^

### Noncanonical modulations of EZH2 in human cancer

Apart from methylating histones, EZH2 possesses the ability to methylate a range of non-histone protein substrates as well. EZH2 can physically interact with signal transducer and activator of transcription 3 (STAT3) and methylate STAT3 directly, promoting nuclear retention and increasing the activity of STAT3 and therefore exacerbating cancer.^8^^,^^9^ Other non-histone targets for methylation by EZH2 have been identified, such as GATA binding protein 4 (GATA4), talin, Jarid2, Elongin A, retinoic acid-related orphan receptor alpha, and promyelocytic leukemia zinc finger protein,^10^ which contributes to either transcriptional silencing or transcriptional activation. This also proved that EZH2 can not only silence downstream genes but also have transcriptional activation activity. Accumulating evidence indicates that EZH2 contains a hidden and partially disordered trans activation domain, which directly binds to transcription coactivator p300 and activates gene expression in cancer cells.^11^

EZH2 can also bind to nucleic acids directly, activating gene expression independent of Polycomb. Kim et al reported that EZH2, as a transcriptional activator, directly binds to its promoter to activate the gene expression of androgen receptor in a manner independent of Polycomb and methylation.^12^ Similar action was also found in ovarian cancer stem cells that EZH2 transcriptionally up-regulates checkpoint kinase 1 expression by directly binding to its promoter, therefore promoting ovarian cancer chemoresistance.^13^

## Regulation of EZH2 in human cancer

To further investigate the potential mechanism underlying the overexpression of EZH2, we discovered that EZH2 can be transcriptionally induced by multiple factors such as p53,^14^ MYC,^15^ ETS-related gene,^16^ E2F transcription factor 7,^17^ and vascular endothelial growth factor.^18^ The overexpression of EZH2 in cancer cells may also be due to the regulation of some RNAs at different levels. Some microRNAs were found to inhibit EZH2 expression as listed in Table 1.

**Table 1:** The microRNAs that regulate EZH2.

MicroRNA	Cancer	Effect
miR-144	Astrocytoma	Forced expression of miR-144 suppressed astrocytoma cell proliferation, invasion, and migration by down-regulating EZH2.^120^
miR-130–5p	Lung adenocarcinoma	High expression of miR-130–5p reduced the cell viability and inhibited cancer cell metastasis and invasion.^121^
miR-625–3p	Gastric cancer	Inhibition of miR-625–3p significantly enhances cell proliferation and invasiveness.^122^
miR-101–3p	Bladder urothelial carcinoma	miR-101–3p advances the sensitivity of bladder urothelial carcinoma to cisplatin through targeted EZH2.^123^
miR-138	Glioblastoma	Ectopic expression of miR-138 effectively inhibits glioblastoma cell proliferation *in vitro* and tumorigenicity *in vivo* by inducing cell cycle G1/S arrest.^124^
miR-101	Non-small cell lung cancer	miR-101 inhibits cell proliferation and invasion and enhances paclitaxel-induced apoptosis in non-small cell lung cancer cells, at least in part, by directly repressing EZH2 expression.^125^
miR-526b	Non-small cell lung cancer	miR-526b influences the attenuated viability and wound closure ability in A549 cells and migratory ability through targeted EZH2.^20^
miR-124	Myeloid malignancies	miR-124 plays a crucial role in the single-agent epigenetic therapy reaction.^126^
miR-33b	HER2^+^ breast cancer	The inhibition of miR-33b induces cell proliferation, invasion, migration, epithelial–mesenchymal transition, and EZH2 expression in non-tumorigenic cells.^127^
miR-98, miR-214	Esophageal squamous cell carcinoma	Overexpression of miR-98 and miR-214 inhibited the migration and invasion of esophageal squamous cell carcinoma cells.^128^

EZH2, enhancer of zeste homolog 2; HER2, human epidermal growth factor receptor 2.

Although numerous microRNAs have been demonstrated to target and down-regulate EHZ2 expression, the expression levels of these microRNAs are typically reduced in tumor tissue compared with normal tissue. The observed down-regulation of microRNAs and up-regulation of EZH2 mRNA in various cancers is associated with clinical features such as pathological grading and tumor staging. Also, evidence suggests that miRNAs may be adsorbed and inhibited by other types of RNA through the sponge effect, maintaining high expression of EHZ2 in tumors.

Long-chain non-coding RNA (lncRNA) can regulate gene expression at multiple levels and is related to the function of EZH2 and its maintenance of high expression in tumor cells. The up-regulation of lncRNA plasmacytoma variant translocation 1 expression is closely related to the occurrence, progression, and prognosis of non-small cell lung cancer.^19^ Further study found that PTV1 silenced miRNA-526b which is the upstream regulated miRNA of EZH2, maintaining the expression of EZH2.^20^ lncRNA nuclear-enriched abundant transcript 1 could up-regulate the expression of EZH2 by targeting miR-144–3p, promoting the function of EZH2 in the progression of endometrial cancer.^21^

EZH2 can also be recruited to the target gene by a number of lncRNAs, such as LINC00152^22^ and LINC01559,^23^ to exert its biological function. Recent studies have shown that lncRNA can be used as a molecular scaffold to help epigenetic enzymes bind to EZH2 promoters.^24^ It is corroborated by chromatin immunoprecipitation assay that lncRNA small nucleolar RNA host gene 8 intensified the enrichment of EZH2 and H3K27me3 in the promoter region of reversion-inducing cysteine-rich protein with Kazal motifs, leading to human papillomavirus-induced cervical carcinoma aggravation.^25^ Besides, EZH2 can also be stabilized by the ubiquitination of lncRNA.^26^ Some lncRNAs that contribute to EZH2's function are listed in Table 2.

**Table 2:** The lncRNAs that regulate EZH2.

lncRNA	Cancer	Effect
*lncRNA sponges upstream miRNA of EZH2*		
TUG1	Pancreatic carcinoma	TUG1 competitively sponges miR-382 to regulate EZH2 and promote tumor progression.^129^
NEAT1	Endometrial cancer	NEAT1 acts as a competitive endogenous RNA of miR-144–3p, leading to EZH2 up-regulation and promoting cell proliferation and invasion.^21^
MALAT1	Colorectal cancer	MALAT1 promotes EZH2 expression and the development of colorectal cancer as a competitive endogenous RNA by sponging miR-363–3p.^130^
ADAMTS9-AS2	Tongue squamous cell carcinoma	ADAMTS9-AS2 promotes tongue squamous cell carcinoma proliferation and metastasis by sponging miR-600, enhancing EZH2 enhancer expression.^131^
SPRY4-IT1	Bladder cancer	SPRY4-IT1 acts as miR-101–3p sponge to positively regulate EZH2 expression, thus promoting bladder cancer pathogenesis.^132^
LINC00662	Oral squamous cell carcinoma	LINC00662 directly targets miR-144–3p to up-regulate EZH2 and accelerate oral squamous cell carcinoma progress.^133^
DLGAP1-AS1	Glioma	DLGAP1-AS1 promotes glioma progression by regulating EZH2 expression through sponging miR-1297.^134^
*EZH2 is recruited by lncRNA*		
SNHG8	Cervical carcinoma	SNHG8 recruited EZH2 to down-regulate RECK expression, leading to the aggravation of human papillomavirus-induced cervical carcinoma.^25^
CASC9	Non-small cell lung cancer	CASC9 promotes gefitinib resistance by recruiting EZH2 to inhibit the expression of dual specificity phosphatase 1 epigenetically.^135^
LINC00152	Esophageal cancer	LINC00152 promotes the resistance of esophageal cancer cells to oxaliplatin and epithelial–mesenchymal transition by recruiting EZH2.^22^
LINC01559	Gastric cancer	LINC01559 recruits EZH2 to repress PTEN.^23^
FOXP4-AS1	Gastric cancer	FOXP4-AS1 combines with EZH2/lysine-specific demethylase 1 to form a carcinogenic complex, accelerating gastric cancer cell proliferation, migration, and invasion.^136^
HOTAIR	Nasopharyngeal carcinoma	HOTAIR promotes nasopharyngeal carcinoma cell progression by recruiting EZH2.^137^
ZEB2-AS1	Non-small cell lung cancer	ZEB2-AS1 aggravates the malignant progression of non-small cell lung cancer by recruiting EZH2 to negatively regulate PTEN.^138^
CASC9	Esophageal squamous cell carcinoma	CASC9 promotes esophageal squamous cell carcinoma cell growth by recruiting EZH2 and negatively regulating programmed cell death 4 expression.^139^
LINC00673	Papillary thyroid carcinoma	LINC00673 inhibits p53 expression by combining with EZH2 and DNA methyltransferase 1, promoting papillary thyroid carcinoma cell proliferation and invasion.^140^
FOXC2-AS1	Melanoma	FOXC2-AS1 inhibits p15 transcription via recruiting EZH2 to stimulate the cell proliferation of melanoma.^141^
*EZH2 is stabilized by lncRNA*		
FAM83C-AS1	Colorectal cancer	FAM83C-AS1 combines with ZRANB1 to promote EZH2 deubiquitination, stabilizing EZH2 protein expression.^26^

EZH2, enhancer of zeste homolog 2; TUG1, taurine upregulated gene 1; PTEN, phosphatase and tensin homolog; NEAT1,nuclear enriched abundant transcript 1; MALAT1, metastasis associated lung adenocarcinoma transcript 1; ADAMTS9-AS2, ADAMTS9 antisense RNA 2; SPRY4-IT1, SPRY4 intronic transcript 1; DLGAP1-AS1, DLGAP1 antisense RNA 1; CASC9, cancer susceptibility candidate 9; FOXP4-AS1, Forkhead box P4 antisense RNA 1; HOTAIR, HOX antisense intergenic RNA; ZEB2-AS1, Zinc finger E-box binding homeobox 2 (ZEB2) antisense RNA 1; FOXC2-AS1, Forkhead box protein C2 (FOXC2) antisense RNA 1; FAM83C-AS1, FAM83C antisense RNA 1; ZRANB1, zinc finger RANBP2-type containing 1.

Circular RNA (circRNA) is a bioactive nucleic acid molecule that exists in the form of closed-loop RNA and plays a key role in the occurrence, development, and pathogenesis of various cancers and is reported to participate in cancer progression by regulating EZH2 as a molecular sponge as shown in Table 3.^27^, ^28^, ^29^, ^30^, ^31^, ^32^, ^33^, ^34^, ^35^ In addition, EZH2 can be recruited by circRNAs to execute its function.^36^, ^37^, ^38^ circ-LRIG3 forms a ternary complex with EZH2 and STAT3 in hepatocellular carcinoma to promote the methylation and subsequent phosphorylation of STAT3 induced by EZH2, thus activating STAT3 signal transduction.

**Table 3:** The circRNAs that regulate EZH2.

circRNA	Cancer	Effect
*circRNA sponges upstream miRNA of EZH2*		
circ_0071589	Colorectal cancer	circ_0071589 promotes EZH2 expression by sponging miR-600, promoting colorectal cancer growth, invasion, and migration.^27^
circ_0115744	Colorectal cancer	circ_0115744, as a competitive endogenous RNA of miR-144, weakens the inhibitory effect on its target EZH2.^28^
circ_PRMT5	Non-small cell lung cancer	circ_PRMT5 simultaneously sponges miR-377, miR-382, and miR-498, attenuating their inhibition of EZH2.^29^
circ_PRDM2	Osteosarcoma	circ_PRDM2 regulates EZH2 positively by sponging miR-760, promoting the resistance of osteosarcoma to doxorubicin.^30^
circ_0020123	Non-small cell lung cancer	circ_0020123 up-regulates EZH2 through competitive binding with miR-144.^31^
circ-TRPS1	Pancreatic carcinoma	circ-TRPS1 enhances EZH2 expression by sponging miR-124–3p, promoting pancreatic carcinoma progression.^32^
circ_ANKIB1	Osteosarcoma	circ_ANKIB1 binds to miR-26b-5p and regulates EZH2, accelerating the chemical resistance of osteosarcoma.^33^
circ_0026123	Ovarian cancer	circ_0026123 promotes EZH2 expression through sponging miR-124–3p.^34^
circ_SYPL1	Hepatocellular carcinoma	circ_SYPL1 sponges miR-506–3p to increase EZH2 expression.^35^
*circRNA binds to EZH2*		
circ-LRIG3	Hepatocellular carcinoma	circ-LRIG3 form ternary complexes with EZH2 and STAT3 that promotes EZH2-induced STAT3 methylation and subsequent phosphorylation, leading to activation of STAT3 signaling.^36^
circ_000623	Laryngeal squamous cell cancer	circ_ 0006232 promotes EZH2 expression through interaction with Fused in sarcoma.^37^
circ_0019435	Cervical cancer	EZH2 was recruited to inhibit the transcription of dickkopf1 and phosphatase and tensin homolog, promoting cervical cancer cell proliferation, invasion, and epithelial–mesenchymal transition.^38^

EZH2, enhancer of zeste homolog 2; STAT3, signal transducer and activator of transcription 3.

### Regulation of EZH2 by post-translational modification

Phosphorylation, one of the most well-studied post-translational modifications of EZH2, is essential in adjusting the role of EZH2. Some studies have suggested that the overexpression of EZH2 may be not sufficient to drive tumorigenesis, and the phosphorylation of EZH2 is a prerequisite for the development of some tumors. Phosphorylation of EZH2 at serine 21 was found to be highly expressed in stem-like cells of glioblastoma multiforme, and this phosphorylation by protein kinase B (AKT) signaling facilitates STAT3 methylation by EZH2, thus enhancing STAT3 activity.^9^ In breast cancer, phosphorylation of EZH2 at T416 by cyclin-dependent kinase 2 is sufficient to promote tumor metastasis. The phosphorylation mediated by p38 at T367 promotes EZH2 cytoplasmic localization, which may regulate migration and invasion of cancer cells.^39^ However, AMP-activated protein kinase-mediated phosphorylation of EZH2 at T311 inhibits PRC2 methyltransferase activity to relieve PRC2-dependent epigenetic silencing and subsequently suppresses tumorigenesis.^40^

Furthermore, EZH2 can also be methylated^41^ and acetylated,^42^ which enhances its stability and contributes to its cancer-promoting effect. Proteins targeting EZH2 may also contribute to the stability of EZH2's function. EZH2 was found to have physical interaction with ubiquitin-specific processing protease 7^43^ and is a deubiquitination target of ubiquitin-specific processing protease 7^44^ which increases the EZH2 protein stability.

## The role and function of EZH2 in human cancer

The significance of EZH2 in cancer was first realized in 2002 when Varambally and colleagues elucidated the association between EZH2 and prostate cancer prognosis.^45^ Experiments show that ubiquitously enforced EZH2 expression in mice can induce the occurrence of lung cancer,^46^ indicating its carcinogenicity.

The phenomenon that EZH2 has been observed to be overexpressed in various types of cancers but not in normal tissues attracts people's attention. Usually, the overexpression of EZH2 is associated with poor prognosis and short survival time in patients with cancer.^47^ At present, it has been found that EZH2 has a wide range of targets. By silencing or activating target proteins, EZH2 is involved in many links of cancer tumorigenesis and development, such as cell proliferation, epithelial–mesenchymal transition, invasion, and drug resistance of cancer cells, making it an important biomarker for cancer therapy.

### Cancer-promoting effect of EZH2

In non-small cell lung cancer cells, the expression of cyclin D1 was significantly decreased after EZH2 silencing.^48^ Meanwhile, p21, a well-known cell cycle inhibitor, was proved to be down-regulated by EZH2 to proliferate gastric cancer cells.^49^ Inhibition of cell cycle arrest by EZH2 has also been found in other cancers such as cholangiocarcinoma,^50^ multiple myeloma,^51^ and small-cell lung cancer.^52^

Besides regulating the cell cycle directly, EZH2 can promote cancer development by participating in multiple signaling pathways. It has been found to activate the PI3K/Akt/mTOR pathway in a variety of cancer cells, such as gastric cancer cells,^53^ glioblastoma cells,^17^ and non-small cell lung cancer cells.^54^ The PI3K/Akt/mTOR signaling pathway is closely related to cell survival and growth and is often enhanced in tumor cells. Vascular endothelial growth factor A, a highly specific vascular endothelial growth factor promoting angiogenesis in tumorigenesis, is positively regulated by EZH2 through the PI3K/AKT signaling pathway to promote the growth of non-small cell lung cancer.^55^ In laryngeal cancer, EZH2 targets runt-related transcription factor 3 through the Wnt/β-catenin signaling pathway to regulate cell proliferation.^56^ In addition, EZH2 promotes the proliferation and migration of bladder cancer via the JAK2/STAT3 pathway.^57^ In total, the above research confirmed the cancer-promoting effect of EZH2 in human cancer, indicating that EZH2 acts as an oncogene.

### EZH2 promotes cancer progression

The overexpression of EZH2 in cervical cancer^58^ and head and neck cancer^59^ was negatively correlated with lymph node metastasis by immunohistochemical study. Moreover, it has also been proven to be related to tumor cell proliferation and invasion in gastric cancer,^60^ non-small cell lung cancer,^61^ ovarian carcinoma,^62^ renal cell carcinoma,^63^ and many other cancers. Celina et al found that overexpression of EZH2 in breast epithelial cells induces adhesion-independent growth and cell invasion and promotes tumor transformation of breast epithelial cells, making it a marker of invasive breast cancer.^64^

Mechanistically, overexpression of EZH2 promotes cancer invasion and migration by directly silencing the expression of related genes that impedes cell invasion and migration such as E-cadherin,^65^ Slit homolog 2,^66^ and Forkhead box C1^67^ in a variety of cancers. The adhesion function of E-cadherin, a key component of intercellular adhesion on the cell surface, keeps cells together, enhancing other cell–cell interactions and physically preventing cell movement.^68^^,^^69^ Ectopic expression of EZH2 suppresses the expression of Dickkopf1^70^ and E-cadherin^71^ through H3K27me3, activating the Wnt/β-catenin signaling pathway, therefore accelerating tumor metastasis. In breast cancer, EZH2 may induce focal adhesion kinase/TGF-β signal activation and then promote cell invasion and migration.^72^ Obviously, multiple pathways are involved in the EZH2-promotion of cancer progression.

Additionally, some studies link EZH2 activity to dedifferentiation. In pancreatic ductal adenocarcinoma, EZH2 silences GATA binding protein 6 (GATA6), an epithelial differentiation-related protein, through epigenetic modification, and participates in the transformation of pancreatic ductal adenocarcinoma to a subtype prone to metastasis.^73^ All the above evidence proved the cancer progression promotion effect of EZH2.

### EZH2 regulates cancer cell death

#### EZH2 inhibits cancer cell apoptosis

Apoptosis is a form of programmed cell death. One of the basic changes from normal cells to tumor cells is the escape of apoptosis, leading to carcinogenesis, tumor progression, and therapeutic resistance. Evidence advocates that targeting apoptosis in cancer is feasible.^74^

Tuberous sclerosis 2 is the basic inhibitor of the mechanistic target of rapamycin (mTOR) signaling pathway that can modulate apoptosis. The overexpression of EZH2 significantly inhibits the expression of tuberous sclerosis 2, thereby activating the mTOR signaling pathway.^75^ Inhibition of EZH2 raises the transcription of DEP domain-containing mTOR-interacting protein, and then represses the activities of mTOR complex 1 and mTOR complex 2, resulting in apoptosis.^76^ Liu et al found that EZH2 gene could further interfere with tumor apoptosis by inhibiting the Bax/Bak signaling pathway in non-small cell lung cancer cells.^77^ By suppressing the TGF-β-Smad-ASCL1 pathway, EZH2 overexpression constricted transforming growth factor-beta (TGF-β)-mediated apoptosis and promoted the progression of small cell lung cancer.^78^ Briefly, the inhibition of EZH2 on apoptosis provides a basis for targeting EZH2 to treat cancer.

#### EZH2 participates in the regulation of autophagy

Autophagy, a process in which lysosomes engulf and degrade their cytoplasmic proteins or organelles, is vital for maintaining cellular homeostasis. In the early stage of tumor occurrence, autophagy prevents tumor occurrence and inhibits cancer progression. However, once the tumor progresses to the late stage, autophagy is helpful to the survival and growth of established tumors.^79^

EZH2 also plays a dual character in regulating autophagy. mTOR is a serine/threonine kinase that mainly regulates cellular metabolism, and autophagy is one of its induced effects.^80^ It has been confirmed that EZH2 regulates a group of common target genes, including tuberous sclerosis 2, Ras homolog gene family member A, DEP domain-containing mTOR-interacting protein, FK506-binding protein 11, regulator of G-protein signaling 16, and glycosylphosphatidylinositol, which are essential inhibitors of mTOR signaling pathway thus inhibiting autophagy.^75^ EZH2 is considered to be positively correlated with autophagy-related protein LC3 in laryngeal squamous cell cancer^81^ and lung cancer,^82^ implying its possibility to promote autophagy. The upstream role of EZH2 in autophagy regulation also proves the potential application of targeted EZH2 in tumor therapy.

#### EZH2 inhibits tumor cell ferroptosis

Ferroptosis, an iron-dependent, new programmed cell death mode, is different from apoptosis, cell necrosis, and autophagy. Inducing ferroptosis is expected to become a new method to kill cancer cells and restrain cancer growth.^83^ Targeting EZH2 may also induce ferroptosis in tumor cells. Studies have shown that EZH2 can inhibit ferroptosis in tongue squamous cell carcinoma cells by regulating miR-125b-5p and key regulatory proteins of ferroptosis such as recombinant solute carrier family 7 member 11.^84^ With a growing number of research on ferroptosis, increasing evidence is believed to be provided to show the therapeutic role of EZH2 in treating human cancer.

### Targeting EZH2 overcomes cancer therapy resistance

#### Reversal of chemotherapy resistance by targeting EZH2

Chemotherapy plays a critical role in clinical cancer treatment. However, the frequent emergence of drug resistance has seriously affected their anti-cancer efficacy and EZH2 was demonstrated to take part in this process.

Immunohistochemical analysis of EZH2 performed on tumor samples of 360 patients with stage IIIB and IV non-small cell lung cancer who received platinum-based chemotherapy continuously showed that patients with advanced non-small cell lung cancer with EZH2 positive expression showed resistance to cisplatin-based chemotherapy.^85^ Also, it has been reported that in multiple types of cancer, targeting EZH2 can overcome anti-cancer drug resistance. In small-cell lung cancer, EZH2-mediated H3K27me3 silences the expression of schlafen family member 11, a factor implicated in DNA-damage repair deficiency, leading to chemoresistance, and suppression of EZH2 can reverse the resistance of primary epidermal growth factor receptor wild-type lung cancer cells to gefitinib.^86^ Similarly, targeting EZH2 can modulate the resistance of prostate cancer cells to docetaxel.^87^ In glioblastoma, EZH2 promotes the expression of ATP-binding cassette transporter multi-drug resistance, multi-drug resistance-associated protein, and breast cancer resistance protein to strengthen chemoresistance.^88^ Through epigenetic mechanism, EZH2 suppresses miR-381 expression in breast cancer to promote cisplatin resistance^89^ and suppresses recombinant F-Box protein 32 expression in gastric cancer, leading to 5-fluorouracil resistance.^90^ To sum up, targeting EZH2 is a potential way to reverse chemotherapy resistance in human cancer.

#### Enhancement of radiosensitivity by inhibiting EZH2

Radiation therapy is an important way of tumor treatment, which destroys genetic material through radiation and deprives cancer cells of their potential for proliferation. Evidence suggests that high expression of EZH2 is associated with a higher risk of recurrence of metastatic disease after radiation therapy, and is involved in promoting the radioresistance of prostate cancer cells.^91^ In hepatocellular carcinoma cells, EZH2 knockdown enhances the radiosensitivity of hepatocellular carcinoma cells^92^ and similar conclusions have been verified in pancreatic cancer.^93^ Therefore, inhibiting EZH2 could be a feasible scheme to increase radiosensitivity in human cancer.

#### Improvement of targeted therapy resistance by targeting EZH2

Targeted therapy involves drugs that directly or indirectly attack specific genetic biomarkers found in specific cancers. It includes monoclonal antibodies, small molecule inhibitors, antibody–drug conjugates, and immunotherapy based on immune checkpoint. In the targeted treatment of breast cancer, EZH2-mediated silencing of protein phosphatase 2 regulatory subunit B leads to drug tolerance and acquired resistance to anti-human epidermal growth factor receptor 2 therapy.^94^ In lung adenocarcinoma, the inhibition of EZH2 reduced the malignant potential of lung adenocarcinoma and increased the sensitivity of cells to platinum and vascular endothelial growth factor receptor 2 targeted therapy.^95^

It is well known that anti-programmed cell death-1/programmed cell death-ligand 1 (PD-1/PD-L1) acts as a promising cancer treatment method by regulating the interaction between immune cells and tumor cells. PD-1/PD-L1 inhibitors are used as an effective immunotherapy for treating lots of human cancers. Intriguingly, the inhibition of EZH2 makes mouse prostate tumor sensitive to PD-1 checkpoint inhibitor^96^ and recovers cell surface major histocompatibility class I in K-562 and cell lines representing neuroblastoma, small cell lung cancer, Merkel cell carcinoma,^97^ and diffuse large B-cell lymphoma.^98^ Moreover, targeting EZH2 in head and neck cancer can enhance anti-tumor immunity and overcome anti-PD-1 resistance.^99^ In summary, those studies showed a very promising strategy to improve targeted therapy resistance by suppressing EZH2.

### EZH2 inhibition enhances anti-cancer immunity

Immunotherapy is a rising treatment in tumor therapy, while tumor immune escape is a major obstacle to immunotherapy. As mentioned above, EZH2 inhibition could enhance anti-tumor immunity and is effective in improving immunotherapy, showing its property in tumor immune escape and immunosuppressive function.

Studies indicate that EZH2 can regulate the polarization of immune cells by directly binding to transcription factors. Immunofluorescence analysis showed that EZH2 could be detected in the nucleus and cytoplasm of activated T cells.^100^ EZH2 strongly binds to t-box transcription factor protein 21 and GATA binding protein 3 (GATA3), the main regulators of T-helper 1 (Th1) and T-helper 2 (Th2) cell differentiation respectively and regulates Th1 and Th2 polarization.^101^ In CD4^+^ T cells, EZH2 targets transcription factors T-bet, GATA3, and retinoic acid-related orphan receptor alpha, suppressing the polarization of Th1, Th2, and T-helper 17.^102^ However, the number of Forkhead box P3 CD4 cells in EZH2-deficient mice decreased *in vivo*, which is correlated with the impairment of Treg cell function.^103^ In natural killer cells, EZH2 may directly regulate the expression of transcription factor pre-B cell leukemia transcription factor 1 which is reported to promote natural killer cell development.^104^

In addition, EZH2 can affect immune cell function by adjusting quantities of chemokines. PRC2 controls T cell trafficking and affects colon cancer pathology by inhibiting Th1 type chemokines C-X-C motif chemokine ligand 9 and C-X-C motif chemokine ligand 10.^105^ Meanwhile, EZH2 inhibition enhanced natural killer cell-mediated tumor growth inhibition by re-expressing C-X-C motif chemokine ligand 10 and enhancing natural killer cell migration and recruitment to tumor sites.^106^ In B-cell lymphoma, EZH2 inhibition can increase the expression of the chemokine C–C motif chemokine ligand 17, induce T-cell chemotaxis, and promote the T-cell-rich tumor microenvironment.^107^ In small-cell lung cancer, the EZH2/H3K27me3 axis silences C–C motif chemokine ligand 2 and suppresses macrophage infiltration, thus promoting tumor development.^108^ It was identified that EZH2-deficient *CD4*^*+*^ T cells produce significantly more interferon-γ, further supporting that EZH2 can inhibit the function of immune cells.^103^

**Figure. 1:**
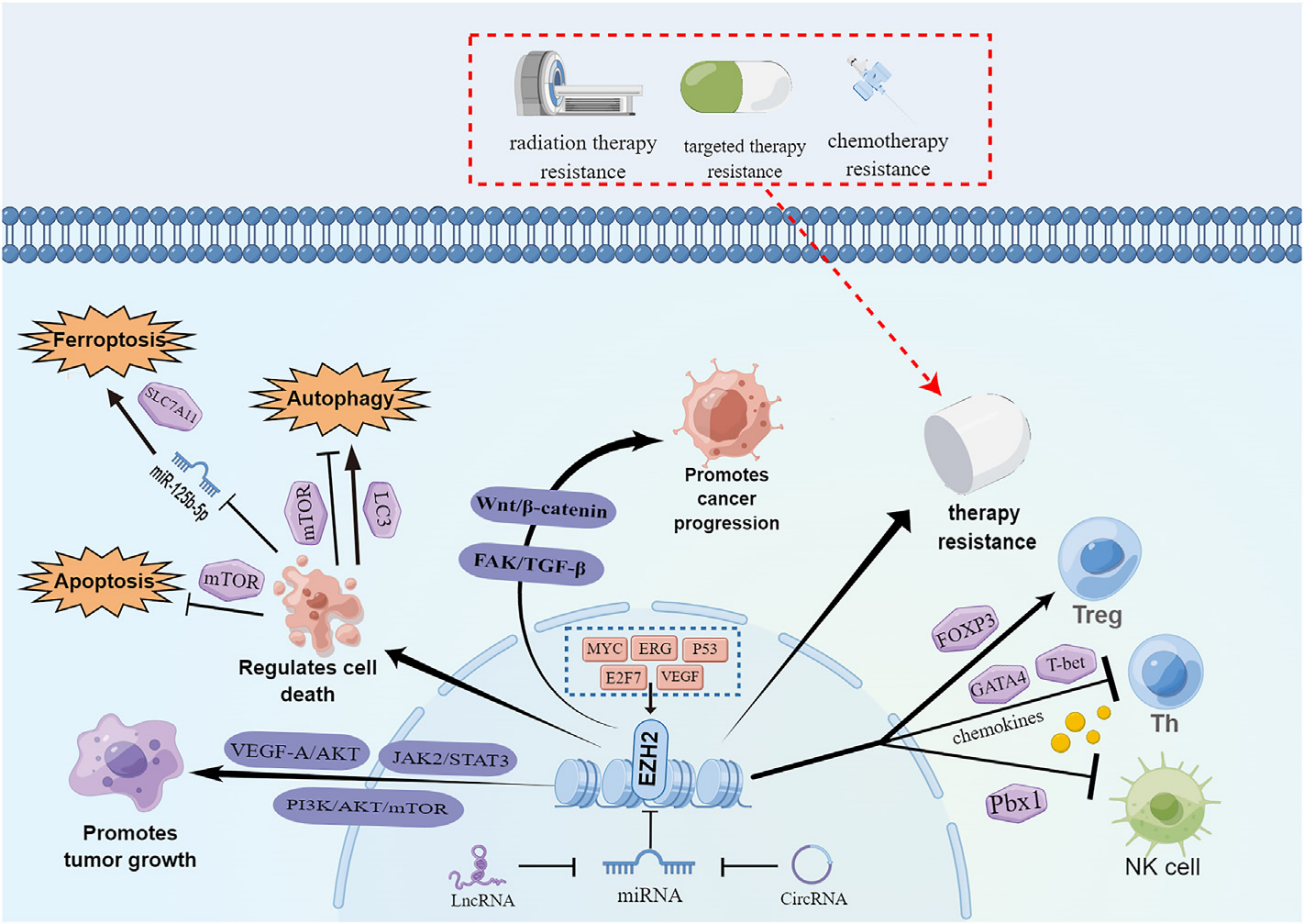
Roles of EZH2 in cancer. i) Up-regulated by transcription factors and RNAs, EZH2 participates in many aspects of tumor progression by regulating multiple proteins and pathways, acting as an oncogene. ii) Through the JAK2/STAT3, PI3K/Akt/mTOR, and VEGF-A/AKT pathways, EZH2 promotes tumor growth. By modulating the Wnt/β-catenin and FAK/TGF-β pathways, EZH2 promotes cancer progression. By regulating mTOR, LC3, and miR-125b-3p, EZH2 regulates cancer cell death. Also, EZH2 is claimed to take part in cancer therapy resistance. iii) In tumor immunity, EZH2 interferes with the functions of T cells and NK cells by represses the expression of transcription factors and chemokines, and may maintain Treg immunosuppressive function through FOXP3. JAK2, Janus Kinase 2; STAT3, signal transducer and activator of transcription 3; PI3K, phosphatidylinositol-3-kinase; AKT, protein kinase B; mTOR, mechanistic target of rapamycin; VEGF-A, vascular endothelial growth factor A; FAK, focal adhesion kinase; TGF-β, transforming growth factor-beta; NK, natural killer; FOXP3, Forkhead box P3; GATA4, GATA binding protein 4; Pbx1, pre B cell leukemia transcription factor 1; Treg, regulatory T cell; Th, T-helper cell; SLC7A11, solute carrier family 7, member 11. The figure was made by Figdraw.

In conclusion, EZH2 is involved in a wide range of tumor biological behaviors, including promoting the survival and proliferation of cancer cells, promoting the migration and invasion of cancer cells, playing a key role in drug resistance in cancer cells, and regulating tumor immunity (Fig. 1). Therefore, targeting EZH2 will become an effective anti-tumor strategy.

### Targeting EZH2 in clinical practice

Based on the critical role of EZH2 in tumors, targeting EZH2 has become a research hotspot in recent years. Aiming at epigenetic modification of EZH2, the earliest EZH2 inhibitor was 3-Deazaneplanocin A. It was reported that 3-Deazaneplanocin A selectively inhibited the H3K27me3 and lysine 20 on histone H4, and reactivated silenced genes in cancer cells.^109^ On January 23, 2020, the biopharmaceutical company Epizyme announced that the Food and Drug Administration would accelerate the approval of tazveraik (tazemetostat) to be listed for the treatment of adults with metastatic/locally advanced epithelioid sarcoma and pediatric patients aged 16 and over who are not suitable for complete resection. Tazemetostat is a SAM-competitive inhibitor that has been proven to be safe and effective.^110^

Since EZH2 has an effect independent of PRC2, direct degradation of EZH2 may be a more effective choice. E3 ubiquitin ligase F-box and WD repeat domain-containing protein 7 can make EZH2 unstable and accelerate its ubiquitin-dependent degradation.^111^ Zhongwei Li et al reported that lncRNA anti-differentiation non-coding RNA increased the phosphorylation intensity of thr-345 and thr-487 sites of EZH2, promoted the ubiquitination of EZH2, and subsequently its degradation.^112^ Up to today, many EZH2 inhibitors are undergoing clinical research, as listed in Table 4.

**Table 4:** The clinical studies of EZH2 inhibitors.

ClinicalTrials.gov ID	Study Title	Conditions	Drug	Phase
*Clinical studies of drugs targeting EZH2*				
NCT03603951	A phase 1 study of SHR2554 in subjects with relapsed or refractory mature lymphoid neoplasms	Relapsed or refractory mature lymphoid neoplasms	SHR2554	1
NCT02395601	A study evaluating CPI-1205 in patients with B-cell lymphomas	B-cell lymphoma	CPI-1205	1
NCT04104776	A study of CPI-0209 in patients with advanced solid tumors and lymphomas	Advanced solid tumor, diffuse large B-cell lymphoma, T-cell mesothelioma malignant prostatic neoplasms, castration-resistant prostate cancer	CPI-0209	1/2
NCT04842877	Study of valemetostat tosylate as a single agent in patients with relapse/refractory B-cell lymphoma	Lymphoma, B-cell lymphoma	Valemetostat tosylate	2
NCT02082977	A study to investigate the safety, pharmacokinetics, pharmacodynamics, and clinical activity of GSK2816126 in subjects with relapsed/refractory diffuse large B cell lymphoma, transformed follicular lymphoma, other non-Hodgkin's lymphomas, solid tumors, and multiple myeloma	Cancer neoplasms	GSK2816126	1
NCT03460977	PF-06821497 treatment of relapsed/refractory small cell lung cancer, castration resistant prostate cancer, and follicular lymphoma	Small cell lung cancer, follicular lymphoma, castration resistant prostate cancer	PF-06821497	1
NCT02900651	Safety and efficacy of MAK683 in adult patients with advanced malignancies	Diffuse large B-cell lymphoma	MAK683 (EED inhibitor)	1/2
*Clinical studies of combination of EZH2 inhibitor with other drugs*				
NCT04762160	SYMPHONY-2, a trial to examine combination of tazemetostat with rituximab in subjects with relapsed/refractory follicular lymphoma	Follicular lymphoma	Tazemetostat, rituximab	2
NCT04557956	Testing the addition of the anti-cancer drug, tazemetostat, to the usual treatment (dabrafenib and trametinib) for metastatic melanoma that has progressed on the usual treatment	Clinical stage IV cutaneous melanoma AJCC v8, metastatic melanoma	Dabrafenib, mesylate, tazemetostat hydrobromide, trametinib dimethyl Sulfoxide	1/2
NCT05205252	A study of tazemetostat in combination with various treatments in participants with blood cancer. (ARIA)	Relapsed hematologic malignancy, refractory hematologic malignancy	Tazemetostat, tafasitamab, lenalidomide, acalabrutinib, daratumumab, mosunetuzumab, pomalidomide, dexamethasone	1/2
NCT04179864	CELLO-1, study of tazemetostat with enzalutamide or abiraterone/prednisone in subjects with castration resistant prostate cancer who have not received chemotherapy	Metastatic pancreatic carcinoma	Tazemetostat, abiraterone/prednisone, enzalutamide	1/2
NCT03854474	Testing the addition of tazemetostat to the immunotherapy drug, pembrolizuma (MK-3475), in advanced urothelial carcinoma	Locally advanced urothelial carcinoma, metastatic urothelial carcinoma, stage III bladder cancer AJCC v8, stage IV bladder cancer AJCC v8	Pembrolizumab, tazemetostat	1/2
NCT03028103	Open-label, multicenter, two-part, phase 1 study to characterize effects of a moderate CYP3A inhibitor on PK of tazemetostat, effects of tazemetostat on PK of CYP2C8 and CYP2C19 substrates, and effect of increased gastric pH on PK of tazemetostat in B-cell lymphoma or advanced solid tumor patients	Diffuse large B cell lymphoma, primary mediastinal lymphoma, marginal zone lymphoma, mantle cell lymphoma, advanced solid tumor	Tazemetostat, fluconazole, omeprazole, repaglinide	1
NCT04224493	Study of tazemetostat in combination with lenalidomide and rituximab compared to taxemetostat with placebo in participants with relapsed/refractory follicular lymphoma (SYMPHONY-1)	Relapsed/refractory, follicular lymphoma	Tazemetostat, lenalidomide, rituximab	3
NCT03480646	ProSTAR: a study evaluating CPI-1205 in patients with metastatic castration resistant prostate cancer	Metastatic castration resistant prostate cancer	CPI-1205, enzalutamide, abiraterone/prednisone	1/2
NCT03525795	ORIOn-E: a study evaluating CPI-1205 in patients with advanced solid tumors	Advanced solid tumors	CPI-1205, ipilimumab	1
NCT04407741	Phase Ⅰ/Ⅱ study of SHR2554 in combination with SHR1701 in patients with advanced solid tumors and B-cell lymphomas	Solid tumor lymphoma	SHR2554, SHR1701	1/2
NCT03879798	DS-3201b and irinotecan for patients with recurrent small cell lung cancer	Small cell lung cancer	DS-3201b, irinotecan	1/2

EZH2, enhancer of zeste homolog 2; PK, pharmacokinetics; AJCC, American Joint Committee on Cancer.

Although targeting EZH2 plays an important role in inhibiting tumors in theory, the therapeutic potential of inhibiting EZH2 in the clinic remains to be explored. Increasing evidence shows that inhibition of EZH2 alone has limited anti-cancer effects in the clinic.^113^, ^114^, ^115^ In an open-label, multicenter, dose-increasing, phase 1 clinical study, 8 of 21 patients with B-cell non-Hodgkin lymphoma (38%) and 2 of 43 solid tumor patients (5%) were observed persistent objective responses, including complete responses.^116^ It was proposed by Xun Huang et al that the inhibitory effect of interfering with EZH2 on solid tumor cell lines is not as significant as that of hematologic tumor cell lines in their study. This resistance can be explained by oncogenic transcriptional reprogramming driven by up-regulation of acetylation at H3K27 after treatment with EZH2 inhibitors.^113^ Also, the complex role of EZH2 in the tumor microenvironment may explain its unsatisfactory clinical benefits. Shu Huang et al found that EZH2 inhibitor GSK126 did not affect the tumors of immunocompetent hosts, which was different from that observed in immunocompromised hosts, indicating that GSK126 leads to immunosuppression.^117^ These results also provide ideas for better clinical application of EZH2 inhibitors — combined administration. EZH1/2 double inhibitor has a more outstanding anti-tumor effect than EZH2 selective inhibitor^114^; gemcitabine/5-fluorouracil plus GSK126 combination therapy improves the efficacy of GSK126 and blocks tumor growth^117^; combined anti-C-C motif chemokine ligand 2 therapy may improve the efficacy of EZH2 inhibitors in breast cancer treatment.^118^ EZH2 inhibitor GSK343 combined with gefitinib also reverses gefitinib resistance in lung cancer.^119^ In Table 4, we also summarized the current clinical studies of EZH2 inhibitors with other drugs.

## Conclusion

Upon a thorough investigation of EZH2 as a prospective therapeutic target for human cancer, its significance in cancer progression has been meticulously investigated and enhanced, earning its recognition as a prime focus in tumor therapy. Acknowledging that EZH2 possesses extensive cancer-promoting capabilities, a primary objective of future clinical and preclinical research lies in exploring the utilization of EZH2 inhibitors, aiming to maximize therapeutic outcomes by precisely targeting EZH2.

## Author contributions

Sumei Wang conceptualized the manuscript, supervised the design and writing of the manuscript, and obtained the funding for this publication. Jun Pan contributed to manuscript editing. Mengfei Xu wrote the manuscript. Chunyan Xu selected the reviewed literature. Rui Wang compiled the review and figure. Qing Tang, Qichun Zhou, Wanyin Wu, Xinliang Wan, and Handan Mo edited the figure and tables.

## Funding

This work was supported by grants from the Research Fund for Bajian Talents of Guangdong Provincial Hospital of Chinese Medicine (China) (No. BJ2022KY13), the National Natural Science Foundation of China (No. 82274602), the Foundation for Basic and Applied Research of Guangdong Province, China (No. 2021A1515220023, 2017B030314166), the Chinese Medicine Science and Technology Research Project of Guangdong Provincial Hospital of Chinese Medicine (China) (No. YN2019QJ06), the Guangzhou Science and Technology Plan Project (Guangdong, China) (No. 2024A03J0548, 202201020349), the Key Project of State Key Laboratory of Dampness Syndrome of Chinese Medicine Jointly Built by Province and Ministry (China) (No. SZ2021ZZ38), Guangdong Provincial Key Laboratory of Chinese Medicine for Prevention and Treatment of Refractory Chronic Diseases (China) (No. YN2023MB09), and the Guangdong Traditional Chinese Medicine Project (China) (No. 20231094, 20241135).

## Conflict of interests

The authors declared no conflict of interest regarding the publication of this paper.
