# Supplementary material for: Treating human cancer by targeting EZH2
Source: Genes Dis. 2024 Apr 25;12(3):101313. doi: 10.1016/j.gendis.2024.101313 (PMC11870178; doi:10.1016/j.gendis.2024.101313)
Supplement: Multimedia component 1 [file mmc1.docx]

Summary of “Targeted EZH2 in human cancer”(**GENDIS-D-23-01306**)

Cancer, as one of the serious diseases threatening human health, has always been a hot spot for researchers. EZH2, an epigenetic regulator usually overexpressed in caner and participates in many processes such as cancer occurrence and development, invasion and migration, drug resistance and anti-tumor immunity as an oncogene, making it an important biomarker in cancer therapy. On January 23, 2020, epizyme company announced that FDA would accelerate the approval of tazveraik (tazemetostat), which was also the first EZH2 inhibitor approved for marketing. This has also further aroused people's attention to targeting EZH2 in cancer. As the diverse functions of EZH2 are discovered, accurately understanding its complexity and diversity functions is a prerequisite for targeting EZH2 in the treatment of cancer. In our work, we summarized the structure of EZH2, gave explanations about what maintains the high expression of EZH2 in cancer, and how it worked in the progression of cancer.

The canonical way for EZH2 to perform its function is to trimethylated histone H3 leading to gene silencing. As a subunit of PRC2, EZH2 catalyzes H3K27me3 in the nucleus and then PRC1 (polycomb repressive complex 1) binds to monoubiquitinated histone H2A at lysine 119 (H2AK-119ub1) and H3K27me3. This complex mediates chromatin compaction followed by transcriptional repression of downstream genes and then involves in maintaining the characteristics of stem cells, regulating gene expression, cell cycle, cell differentiation and the development of tissues and organs. Additionally, studies have found that EZH2 can methylate non histones and affect downstream protein’s function. EZH2 can physically interact with signal transducer and activator of transcription 3 (STAT3) and methylate STAT3 directly, promoting nuclear retention and increasing the activity of STAT3 and therefore exacerbating cancer. Other non-histone targets for methylation by EZH2 have been identified, such as GATA4 (GATA binding protein 4), talin, Jarid2, Elongin A (EloA), RORα (retinoic acid-related orphan receptor alpha) and PLZF (promyelocytic leukemia zinc finger protein), which contributes to either transcriptional silencing or transcriptional activation. Also, EZH2 can act as a transcription factor to directly promote the expression of downstream proteins.

From a mechanistic perspective view, some transcription factors, such as p53, MYC, ERG, E2F7 and VEGF can directly up-regulated the expression of EZH2 in cancer. Some lncRNAs and CircRNAs can act as molecular sponge or molecular scaffold to maintain the high expression of EZH2 and some post-translational modifications of EZH2 contribute to its carcinogenic effect, laying the foundation for its role in cancer.

The function of EZH2 embodies in many aspects of cancer progression. It has been reported that EZH2 promotes tumor progression, invasion, and metastasis in various cancers, and is involved in the regulation of multiple types of cell death, such as cell apoptosis, autophagy, and ferroptosis. In addition, targeting EHZ2 can increase the efficacy of various treatment methods, such as overcoming resistance to chemotherapy and targeted therapy, and increasing sensitivity to radiotherapy. EZH2 also plays an important role in the immune microenvironment. For example, EZH2 can directly affect its function by inhibiting immune cell polarization, or inhibit immune cell infiltration in tumors by inhibiting the expression of chemokines.

Having collected a comprehensive understanding of EZH2’s function in cancer, we considered targeting EZH2 as a theoretically effective way to treat cancer. What correspond to our idea is that some EZH2 inhibitors have already been launched and many are in the clinical trial stage. Based on the different modes of action of EZH2, various EZH2 inhibitors have also been developed, which can be mainly divided into inhibitors of EZH2 methyltransferase activity, inhibitors that break PRC2’s structure and inhibitors triggering EZH2 degradation from a mechanism perspective. However, according to some clinical trial results, inhibiting EZH2 alone did not show satisfactory therapeutic effects. Possible explanations include the special role of EZH2 in the tumor microenvironment and other novel mechanisms. More and more researches are shifting its focus to the combination of EZH2 inhibitors with other drugs. Some ongoing clinical trials are also listed in the main text.

This manuscript was provided by the Clinical and Basic Research Team of Traditional Chinese Medicine in the Prevention and Treatment of Non-Small Cell Lung Cancer form Guangdong Provincial Hospital of Chinese Medicine. Our team mainly studies the efficacy and specific mechanisms of traditional Chinese medicine in the treatment of non-small cell lung cancer, and we have been studying epigenetics in cancer for a long time. EZH2 is also an attractive research direction for our group and we have just published our research findings titled “FZKA reverse gefitinib resistance by regulating EZH2/Snail/EGFR signaling pathway in lung adenocarcinoma”, which was published in the year 2023 of *Journal Of Ethnopharmacology.*
